# Effect of sire population on the genetic diversity and fitness of F1 progeny in the endangered Chinese endemic *Sinocalycanthus chinensis*

**DOI:** 10.1002/ece3.6179

**Published:** 2020-04-03

**Authors:** Junmin Li, Caihong Qi, Jingjing Gu, Zexin Jin

**Affiliations:** ^1^ Zhejiang Provincial Key Laboratory of Plant Evolutionary Ecology and Conservation Taizhou University Taizhou China; ^2^ Traffic Patrol Headquarters of Public Security Bureau of Chongqing City Vehicle Management Institute Chongqin China; ^3^ Xianju Branch of Ecological Environment Bureau of Taizhou City Taizhou China

**Keywords:** genetic diversity, genetic rescue, inbreeding depression, offspring fitness, *Sinocalycanthus chinensis*

## Abstract

*Sinocalycanthus chinensis* Cheng et S. Y. Chang (Calycanthaceae), which has a unique systematic status, is listed as a national second‐class protected plant of China. In this study, the genetic diversity, performance, and fitness of F1 progeny from crosses between the Damingshan (DMS) population of *S. chinensis* and pollen parents from the Daleishan (DLS) and Longxushan (LXS) populations were examined. The DLS population has a relatively small population size, low genetic diversity, and considerable geographical and genetic distances from the DMS population relative to the LXS population. Compared with naturally occurring seeds, DLS‐sired seeds had the highest thousand‐seed weight, starch content, fat content, germination rate, germination index, and emergence rate, but the lowest protein content. Naturally occurring, open‐pollinated seeds had the lowest thousand‐seed weight, starch content, and fat content, but the highest protein content. Compared with natural F1 progeny, DMS × DLS seedlings had the highest genetic diversity, photosynthetic parameters, and growth characteristics, except for leaf mass ratio and stem mass ratio. Under strong light, DMS × DLS seedlings exhibited a *F*_v_/*F*_m_ value of 0.75, while the other two seedling types exhibited *F*_v_/*F*_m_ values of 0.65. DLS‐sired seeds had the most vigorous growth characteristics except for leaf mass ratio and stem mass ratio. These results suggest that genetic rescue by transplanting seedlings from the DLS population or hand pollination with pollen from the DLS population would be effective methods to reduce inbreeding depression and obtain strong offspring with high genetic diversity and fitness in the DMS population.

## Graphical abstract

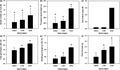

## 1 INTRODUCTION

The term “genetic rescue” is used to describe the improvement of a population's survival rate by introducing genes from another population (Richards, 2000). Genetic rescue is considered a useful tool for the biological conservation of plant populations with low genetic diversity (Ingvarsson, 2001; Pickup, Field, Rowell, & Young, 2013; Richards, 2000) and high genetic differentiation (Ge, Hong, Wang, Liu, & Zhang, 1998). Endangered plants tend to suffer from the fragmentation of their habitats (Jin & Li, 2007; Li & Jin, 2006, 2007). The long‐term persistence of fragmented populations is predicted to be threatened by the loss of genetic variability and increased inbreeding depression (González‐Varo, Albaladejo, Aparicio, & Arroyo, 2010). Thus, genetic rescue is a particularly valuable conservation tool for minimizing extinction risk to endangered plant species (Fenster & Dudash, 1994; Waite et al., 2005).

The supplementation of unrelated genetic materials by genetic rescue can alleviate genetic erosion (Thrall, Richards, McCauley, & Antonovics, 1998) and enhance population viability (Tallmon, Luikart, & Waples, 2004). However, incompatible genetic materials transferred between populations in the process of genetic rescue also have a risk of causing outbreeding depression, resulting in lower fitness and progeny health (Severns, Liston, & Wilson, 2011). Thus, determining the most appropriate sources of genetic material is critical for the successful genetic rescue of endangered plant species (Ingvarsson, 2001; Pickup et al., 2013; Richards, 2000). The genetic characteristics of the source populations contribute substantially to the success of genetic rescue projects (Pickup et al., 2013). Pickup et al. (2013) conducted 2,455 experimental crosses between 12 population pairs of the rare perennial plant species *Rutidosis leptorrhynchoides* (Asteraceae) and found nonlocal genotypes from large populations with high genetic diversity may provide more appropriate genetic sources for restoration compared with local genotypes from small, genetically depauperate populations. It can be concluded that the geographical distance between source and recipient populations and the population size and genetic diversity of the source populations are the most important considerations for genetic rescue (Pickup et al., 2013). However, the role of the genetic characteristics of the genetic materials in determining the efficacy of genetic rescue varies among plant species.

*Sinocalycanthus chinensis* Cheng et S. Y. Chang (Calycanthaceae), commonly known as Chinese sweet shrub (Li & Tredici, 2005), is the only species in the genus *Sinocalycanthus* (Li & Jin, 2006). The size of the natural *S. chinensis* population has contracted, and there remain only a few subpopulations with limited distribution, which are divided geographically into an eastern group and a western group (Zhang, Chen, Qiu, Li, & Jin, 2001; Zhou & Ye, 2002). It has unique systematic status (Li & Jin, 2006) and is listed as one of the national second‐class protected plants of China (Hu, 2002) owing to habitat deterioration caused by tourism development and plant per se overexploitation for its value as a garden ornamental plant (Zhang, 2007). Thus, the conservation of this rare species is urgent (Li & Jin, 2006); however, little is known about the feasibility of utilizing genetic rescue in the conservation of this species.

Population size reductions tend to increase genetic drift and inbreeding, leading to a loss of genetic variation and low genetic diversity of *S. chinensis* at the population level, even though high genetic diversity may be preserved at the species level, through high genetic differentiation among populations (Jin & Li, 2007; Li & Jin, 2006; Li, Jin, & Gu, 2012). *Sinocalycanthus chinensis* is self‐compatible with a mixed mating system that may have evolved from outcrossing to inbreeding (Li & Jin, 2006). Zhang and Jin (2008) found that *S. chinensis* is entomophilous and pollinated by small insects with or without limited flight, and inbreeding depression occurs in *S. chinensis* as a consequence of its limited pollen dispersal. Based on the genetic characteristics of *S. chinensis*, we can hypothesize that genetic rescue would succeed in this species. However, no empirical data have been collected.

Both the genetic diversity and the performance of the progeny have been used separately to evaluate the effect of habitat fragmentation on the fitness of populations in different plant species and regions (González‐Varo et al., 2010; Kolb, 2005; Yates, Elliott, Byrne, Coates, & Fairman, 2007). Although research assessing patterns of progeny performance in conjunction with measures of genetic diversity and mating patterns are rare (but see Broadhurst et al., 2008; Cascante, Quesada, Lobo, & Fuchs, 2002; Mathiasen, Rovere, & Premoli, 2007), assessing plant progeny performance together with measurements of genetic diversity is essential to understanding the efficacy of genetic rescue (González‐Varo et al., 2010). In this study, the genetic diversity, performance, and fitness of F1 progeny from crosses between the Damingshan (DMS) population of *S. chinensis* and pollen parents from the Daleishan (DLS) and Longxushan (LXS) populations were examined. The size and genetic diversity of the DLS and LXS populations and the geographical distances and genetic distances between the DLS and DMS populations and the LXS and DMS populations were compared. We aimed to assess the efficacy of genetic rescue in *S. chinensis*. Specially, we aimed to determine whether the pollen parent population used for genetic rescue had differential effects on genetic diversity, performance and fitness of resulting F1 progeny. These results will enhance the theoretical basis for the genetic rescue of the endangered *S. chinensis*, thus playing a key role in the conservation of this endangered species.

## 2 MATERIALS AND METHODS

### 2.1 Plant species

**Figure 1:**
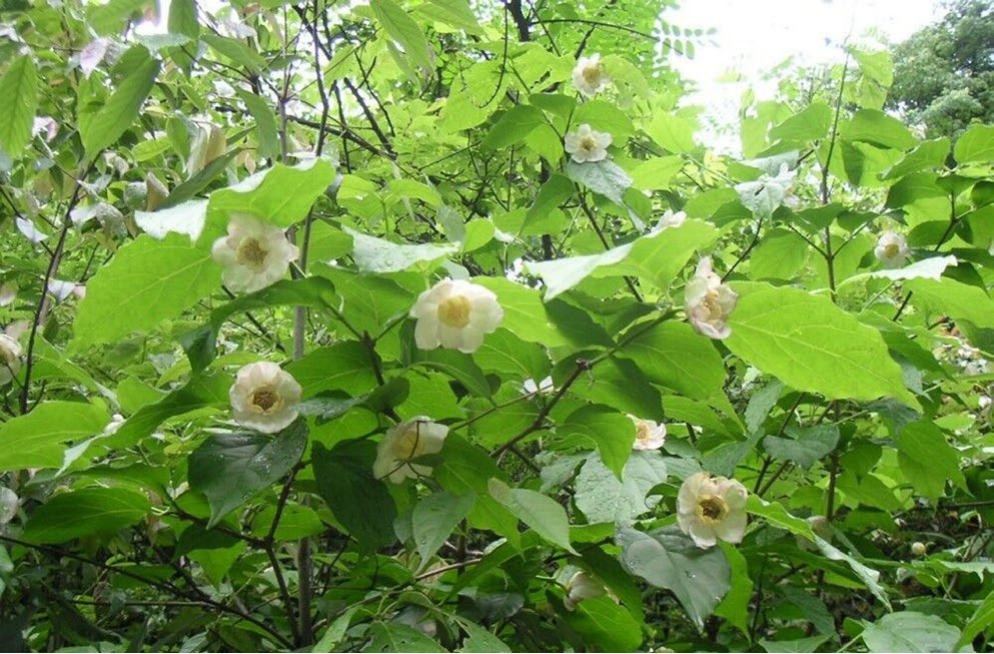
Image of *Sinocalycanthus chinensis*

*Sinocalycanthus chinensis* is a diploid (2*n* = 22) deciduous shrub with glossy green leaves and camellia‐like flowers measuring up to 4″ in diameter (Figure 1). Generally, the height of adult individuals is 6–8 feet. Its broadly elliptic‐to‐ovate leaves are up to 20 cm long and 10 cm wide, growing oppositely and turning from light green to yellow in autumn. Mature plants blooms in June to July. The flowers are creamy white and maroon and lack a noticeable fragrance (Li & Jin, 2006). *Sinocalycanthus chinensis* is an extant representative of a disjunct East Asian–North American genus that is endemic to China. The leaves of *S. chinensis* are used as a remedy for cold, cough, and wheeziness (Ni, Pan, Fu, Wu, & Chen, 2003). The flowers of *S. chinensis* are considered beautiful with high ornamental value (Li & Jin, 2006).

### 2.2 Study site

This study was conducted at Damingshan Mountain (DMS, 30°02′N, 118°59′E), Linan City, Zhejiang Province, China. The DMS population, briefly described in Table 1, is the largest identified population of *S. chinensis*, and it grows within an evergreen broad‐leaved forest near a small stream (Liu, Zhou, Huang, Bao, & Zhao, 2016). The main accompanying species are *Castanopsis eyrei*, *Daphniphyllum macropodum*, *Nyssa sinensis*, and *Dendropanax dentiger*.

**Table 1:** Basic characteristics of different *Sinocalycanthus chinensis* populations

Population	Longitude	Latitude	Altitude (m)	Slope	Number of individuals (#)^a^	Density^a^ (#/hm^2^)	Geographical distance to DMS (km)	Shannon diversity index^b^	Nei's genetic distance to DMS^b^
LXS	118°42′E	30°04′N	912	NE30°	6,243	2,312	27.345	0.0746	0.1027
DLS	120°46′E	28°59′N	782	NE25°	10,360	1,248	209.128	0.0687	0.8160
DMS	118°59′E	30°02′N	854	NE30°	—	—	—	—	—

^a^ The number of individuals and density were collected by Liu et al. (2016).

^b^ Shannon diversity index and Nei's genetic distance were indicated by intersimple sequence repeat (ISSR) molecular markers (Gu, 2010).

### 2.3 Pollen sources

Plants from two *S. chinensis* populations located at two sites with different population sizes, levels of genetic diversity, and relative geographical and genetic distances from the DMS seed parent were used as pollen parents. One is located at Longxushan Mountain (LXS) in Anhui Province, China; this population is small with relatively high genetic diversity and with short geographical and low genetic distances from the DMS population (Table 1). This *S. chinensis* population is also within an evergreen broad‐leaved forest. The main accompanying species are *Litsea coreana* var. *sinensis*, *Symplocos setchuensi,* and *Platycarya strobilacea*. The other pollen parent population is located at Daleishan Mountain (DLS) in Tiantai County, Zhejiang Province, China, and it is a medium‐sized population located a long geographical distance from the DMS population (Table 1). This *S. chinensis* population is located among shrubs within a valley. The main accompanying species are *Camellia cuspidate*, *Spiraea salicifolia*, *Corylopsis sinensis*, *Rhododendron simsii*, *Actinidia chinensis*, and *Sargentodoxa cuneata*. The DLS *S. chinensis* plants occur in the canopy and are intertwined with *A. chinensis* and *S. cuneata*.

### 2.4 Hand pollination and seed collection

Mature *S. chinensis* plants were selected as pollen donors. In May 2009, when the stigmas of *S. chinensis* flowers in DMS had matured, pollens were collected from flowers on 20 mature *S. chinensis* individuals in the LXS and DLS populations using Chinese brushes and stored in sterile plastic tubes. The pollen samples were maintained at 4°C and quickly transported to Damingshan Mountain, where the hand pollination was conducted with the DMS population. Pollen was transferred to DMS stigmas within 6 hr. Thirty mature individual plants in the DMS population were hand‐pollinated with an average of 100 flowers pollinated per treatment. Each plant was randomly assigned to a crossing treatment, and a total of 200 flowers were pollinated. All hand‐pollinated stigmas were saturated with pollen. To exclude natural pollinators, plants were bagged with fine nylon mesh before flowering. Each hand‐pollinated flower was also emasculated before the stigma became receptive to prevent selfing. Pollen was transferred directly from donor flower anthers onto receptive stigmas until the stigmas were saturated. The date was selected to ensure that each flower was encountered at the onset of its stigmatic surface receptivity. Thirty naturally open‐pollinated DMS plants were selected as the control (Bossuyt, 2007; Holmes, James, & Hoffmann, 2008).

### 2.5 Fitness measurement

Fitness of the F1 progeny was defined as the relative reproductive success of a genotype as measured by survival, fecundity, and other life history parameters (Molina‐Montenegro et al., 2013) and was indicated by seed number per fruit, seed weight, seed size germination days, total germination rate, seedling emergence rate (Baker, Richards, & Tremayne, 1994), and seedling biomass (Du, Yang, Guan, & Li, 2016).

In October 2009, *S. chinensis* fruits were collected and air‐dried, and seeds were collected from the dried fruits. Thousand‐seed weights were measured using an electronic balance with an accuracy of 0.0001 g. The starch, lipid, and protein contents of seeds were measured using anthrone–sulfuric acid colorimetric, Soxhlet extractor, and UV‐spectrophotometric methods, respectively (Song, Cheng, Jiang, Long, & Huang, 2008).

In March, 2010, DMS × DLS (pollen) seeds (hereafter, DLSH), DMS × LXS (pollen) seeds (hereafter, LXSH) and control seeds were germinated in an illuminated incubator (Jiangnan Instrument Inc., Ningbo, China) with 30/15°C and 12‐hr/12‐hr light/dark cycles at 80% humidity. Seeds were immersed in H_2_SO_4_ for 3 min, rinsed with sterilized water, and surface sterilized with 70% ethanol. Seeds were immersed in sterilized water and incubated at 28°C for 2 days. Fifty seeds were placed into 3‐mm‐deep silicon sand. Seeds were covered with moist filter paper to prevent them from drying out. Three replicates were used with a total of 150 seeds for each treatment.

Seeds were considered to be germinated when their radicle length exceeded 2 mm (Hussain, Aljaloud, Alshammafy, Karimulla, & Al‐Aswad, 1997), and germination was recorded daily for 100 days. Germination‐related indices were calculated as follows, according to previously described methods (Cai, 2008): (a) germination days were recorded as the number of days after sowing when the seeds begin germinating and (b) total germination rate (%) = total number of germinated seeds on the 100th day/total number of seeds used for the germination experiment × 100%.

In March 2010, *S. chinensis* seeds of F1 DLSH, F1 LXSH, and control progeny were immersed in H_2_SO_4_ for 3 min, rinsed with sterilized water, and surface sterilized with 70% ethanol. Seeds were immersed in sterilized water and incubated at 28°C for 2 days. Seeds were planted 2 cm deep into soil‐filled pots. Three seeds were planted per pot, and a total of 50 pots were used for each treatment. Sufficient tap water was added every day. One hundred days after planting, the seedling emergence rate was defined as the percentage of healthy seedlings that emerged, with a hypocotyl appearing on or above the soil surface (Bolek, 2010; Demir & Mavi, 2004).

After the performance measurement described below, plants were harvested and divided into leaves, stems, and roots. Plant material was oven‐dried at 105°C for 1 hr and then at 80°C until a constant weight was reached. The leaf, stem, and root biomasses were weighed with a balance to an accuracy of 0.1 mg, and total biomass of seedlings was subsequently calculated.

### 2.6 Seedling photosynthetic physiological performance measurement

In May 2010, healthy F1 DLSH, FI LXSH, and control seedlings were transplanted into pots, with each pot containing one seedling. In August 2010, we conducted in situ photosynthetic trait measurements on a sunny day using a mature middle leaflet at the same position across plants, using a GFS‐3000 Portable Gas Exchange Fluorescence System (Walz Heinz GmbH). The photosynthetically active radiation (PAR) was maintained at 800 μmol m^−2^ s^−1^ using a red‐blue LED light source, and the temperature was maintained at 25°C with a relative humidity of 70% inside the leaf measurement chamber. The CO_2_ concentration within the chamber was maintained at 400 µmol/mol. We recorded the net photosynthetic rate (*P*_n_), stomatal conductance (*G*_s_), transpiration rate (*T*_r_), and intercellular CO_2_ concentration (*C*_i_) between 08:30 and 11:30. Three leaves per plant were chosen, and six consecutive measurements were performed (Li, Liao, Guan, Wang, & Zhang, 2012).

To construct light response curves, we obtained all photosynthesis measurements between 09:30 and 11:00 (Beijing time) based on a mature leaf from each plant at a leaf temperature of 25°C, a CO_2_ concentration of 400 ppm, and a relative humidity of 70%. We used a red‐blue LED light source attached to the system to produce steady photosynthetically active radiation (PAR). Prior to the measurements, we allowed the mature leaf to acclimate under a PAR of 2,000 μmol m^−2^ s^−1^ for 30 min to avoid photo‐inhibition. As soon as the value stabilized, we exposed the leaves to a series of PAR values for 20 min or so in the following order: 2,000, 1,500, 1,200, 1,000, 800, 600, 400, 200, 100, 50, 20, and 0 μmol m^−2^ s^−1^. The temporal interval between each concentration was 3 min. We fitted the entire photosynthetic light response curve in Origin 8.0 (OriginLab) as a binary linear equation by calculating the maximum of the net photosynthetic rate (*P*_n_) as *P*max. We utilized the following definitions. The light intensity at the maximum *P*_n_ value (*P*_max_) was defined as the light saturation point (LSP). The light intensity at a zero *P*_n_ value was defined as the light compensation point (LCP). The *P*n at a maximum PAR of zero was defined as the dark respiration point (*R*_d_).

Chlorophyll fluorescence parameters under strong light (1,200 μmol photons m^−2^ s^−1^) were measured between 12:00 and 14:00 with a portable chlorophyll fluorometer (OS30P, Opti‐Sciences Inc.). Measurements were performed on the third undamaged adult leaf from the top of each plant after 30 min of dark adaptation using light exclusion clips. For each measurement, three leaves per plant from three randomly selected plants per measurement were sampled. The data from three measurements of three leaves were averaged and used as the mean for each plant. The variable‐to‐maximum fluorescence ratio (*F*_v_/*F*_m_), which has been used to express the maximum photosystem II (PSII) photochemical efficiency, was calculated.

### 2.7 Seedling morphological performance measurements

In September 2010, plant height, leaf length, and leaf width were measured using a ruler to an accuracy of 0.1 cm, and the leaf length‐to‐width ratio was then calculated. The number of leaves was also recorded. The basal diameter was measured using Vernier calipers to an accuracy of 0.02 cm. Furthermore, whole pots were immersed in water, and then, entire root complexes were excavated and carefully washed with running water to remove fine soil particles. Intact root systems were spread in a Perspex tray (A3‐size) to minimize overlap, scanned (resolution 300 dpi, Epson 1,680, Seiko Epson Corporation, Japan), and analyzed using the WinRhizo software package (Version 3.10, Regent Instruments Inc.) to obtain the root volume (RV), total root length (RL), root surface area (RSA), and number of root tips (RT).

### 2.8 Statistical analysis

The data are presented as mean ± *SD* values. One‐way ANOVA was used to test the significance of the differences among treatments. All the analyses, including principal component analysis (PCA), were conducted using SPSS 19.0 software (IBM Corp.). All graphs were produced using SigmaPlot 13.0 software (Systat Software Inc.).

### 2.9 Genetic diversity of F1 progeny

When the seedlings were 20 days old, 30 healthy seedlings were randomly selected from among the F1 DLSH and LXSH progeny as well as control progeny for genetic diversity analysis using the sequence‐related amplified polymorphism (SRAP) method. One fresh tender leaf from one seedling was crushed into powder in liquid nitrogen, and the genomic DNA was extracted for analysis. Total DNA concentration was determined according to the method described by Li and Jin (2006). Universal SRAP primers were designed according to previously published sequences (Ferriol, Pico, & Nuez, 2003) (Table 2). Primers were synthesized by Shanghai Sangon Biotechnology Inc. SRAP amplification was conducted with the following 10‐μl reaction solution: 1× Taq polymerase buffer, 3.5 mM MgCl_2_, 1 U of Taq DNA polymerase (Promega Inc.), 20 ng of template DNA, 20 mg/ml bovine serum albumin (BSA), 20 pmol of forward primer, 7.5 pmol of reverse primer, and 0.5 mM concentrations of each dNTP. The amplification reaction was performed with a PTC 220 Thermal Cycler (Bio‐Rad Laboratories, Inc.). The touchdown program included an initial 5‐min denaturation at 94°C, followed by 10 cycles of 1 min at 94°C, 1 min at 45°C (touchdown for 1°C every cycle), and 1.5 min at 72°C, followed by 25 cycles of 1 min at 94°C, 1 min at 48.8°C, and 1.5 min at 72°C, with an 8‐min final extension at 72°C.

**Table 2:** Universal SRAP primers used in this study

Forward primer	Reverse primer
me1: 5′TGAGTCCAAACCGGATA‐3′	em1: 5′GACTGCGTACGAATTAAT‐3′
me2: 5′TGAGTCCAAACCGGAGC‐3′	em2: 5′GACTGCGTACGAATTTGC‐3′
me3: 5′TGAGTCCAAACCGGAAT‐3′	em3: 5′GACTGCGTACGAATTGAC‐3′
me4: 5′TGAGTCCAAACCGGACC‐3′	em4: 5′GACTGCGTACGAATTTGA‐3′
me5: 5′TGAGTCCAAACCGGAAG‐3′	em5: 5′GACTGCGTACGAATTAAC‐3′
me6: 5′TGAGTCCAAACCGGTAA‐3′	em6: 5′GACTGCGTACGAATTGCA‐3′
me7: 5′TGAGTCCAAACCGGTCC‐3′	em7: 5′GACTGCGTACGAATTCAA‐3′
me8: 5′TGAGTCCAAACCGGTGC‐3′	em8: 5′GACTGCGTACGAATTCTG‐3′
	em9: 5′GACTGCGTACGAATTCGA‐3′
	em10: 5′GACTGCGTACGAATTCAG‐3′
	em11: 5′GACTGCGTACGAATTCCA‐3′

The PCR amplification products were loaded onto a 1.6% agarose gel (containing 3.5 mg/ml ethidium bromide) with 0.5 × TBE buffer. Electrophoresis was conducted for 1.5 hr at 100 V. After electrophoresis, the gels were visualized with a GIS‐2008 gel imaging and analysis system (Shanghai Tanon Science & Technology Co., Ltd.). The Gel Doc XR system was used for imaging (Bio‐Rad Laboratories). For a negative control, template DNA was replaced with ddH_2_O in the PCR. For every sample, amplification was conducted in triplicate.

Amplified bands were scored across a size range of 0.2–2 kb. SRAP amplified fragments were scored manually for presence (1) or absence (0). The number of polymorphic loci, the percentage of polymorphic loci (*P*), Shannon's information index (*I*, Lewontin, 1972), Nei's gene diversity (*h*), and Nei's (1972) genetic identity and genetic distance between populations were calculated using POPGENE version 1.31 software (Yeh & Boyle, 1997). Unweighted pair group method arithmetic average (UPGMA) cluster analysis was performed to infer the relationships among populations with Nei's genetic distance using POPGENE version 1.31 software (Yeh & Boyle, 1997).

## 3 RESULTS

### 3.1 F1 fitness

**Figure 2:**
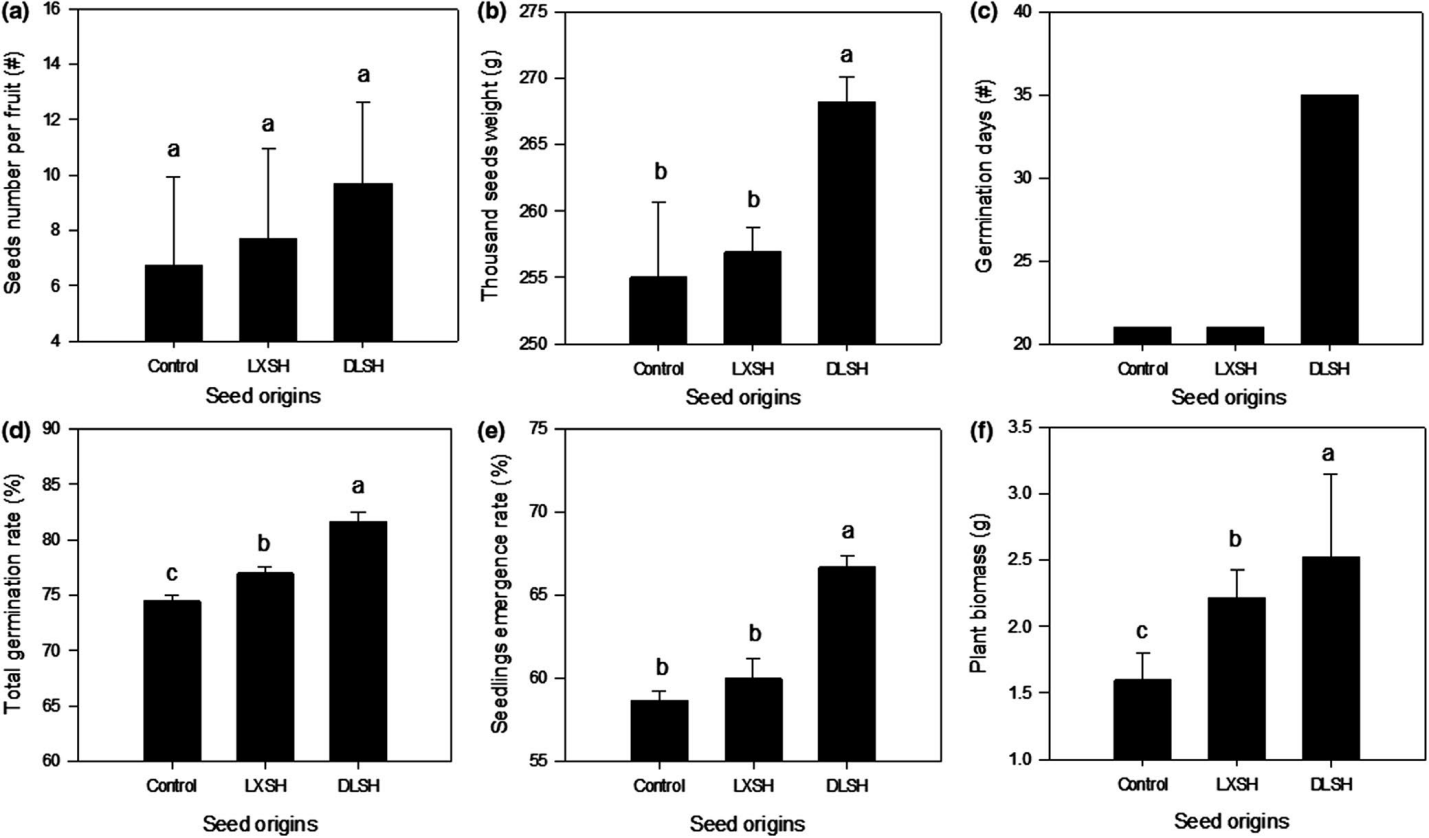
Fitness, including number of seeds per fruit (a), thousand‐seed weight (b), germination days (c), total germination rate (d), seedling emergence rate (e), and plant biomass (f) of F1 progeny of the DMS population hybridized with pollen collected from the DLS population (DLSH) and LXS population (LXSH). Naturally open‐pollinated DMS plants were selected as the control. The data are presented as mean ± *SD* values. Different lowercase letters indicate significant differences between different pollen sources at a *p* < .05 level

**Figure 3:**
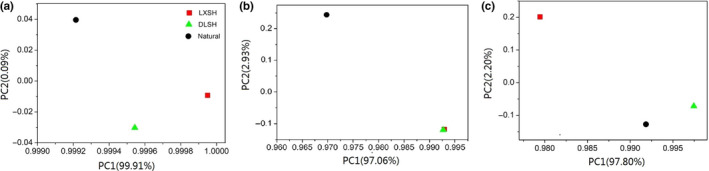
Principal component analysis results based on fitness (a), morphological performance (b), and photosynthetic physiological performance of F1 progeny of the DMS population hybridized with pollen collected from the DLS population (DLSH) and LXS population (LXSH). Naturally open‐pollinated DMS plants were selected as the control

DLSH seeds had the most seeds per fruit (*p* > .05, Figure 2a) and the highest thousand‐seed weight (*p* < .05, Figure 2b). Although they germinated 10 days later than LXSH and control seeds (Figure 2c), DLSH seeds and seedlings had the significantly highest total germination rate, seedling emergence rate, and plant biomass (*p* < .05, Figure 2d–f). LXSH seeds had thousand‐seed weight, number of germination days and seedlings emergence rate values similar to those of the control (Figure 2b–d), but had significantly higher germination rates and plant biomass as seedlings (*p* < .05, Figure 2e,f). PCA revealed that the fitness DLSH seeds and LXSH seeds differed from control seeds, and total germination rate, seedlings emergence rate, and germination days had significant positive contribution to PC1 (Figure 3a).

### 3.2 Morphological performance of seedlings

**Figure 4:**
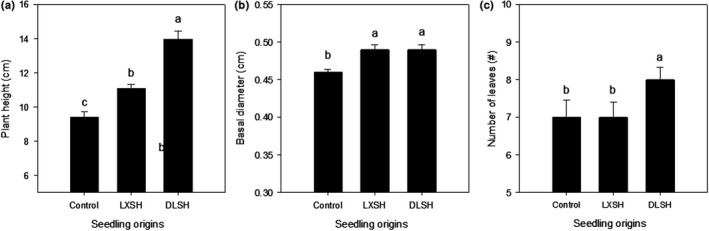
Plant height (a), basal diameter (b), and number of leaves (c) of F1 progeny from the DMS population hybridized with pollen collected from the DLS population (DLSH) and LXS population (LXSH). Naturally open‐pollinated DMS plants were selected as the control. The data are presented as mean ± *SD* values. Different lowercase letters indicate significant differences between different pollen sources at a *p* < .05 level

DLSH seedlings were significantly taller than LXSH seedlings, which were significantly taller than the control seedlings (Figure 4a). DLSH seedlings had a basal diameter similar to that of LXSH seedlings, both of which were significantly higher than that of the control seedlings (Figure 4b). DLSH seedlings had significantly more leaves than the LXSH and control seedlings (Figure 4c).

**Figure 5:**
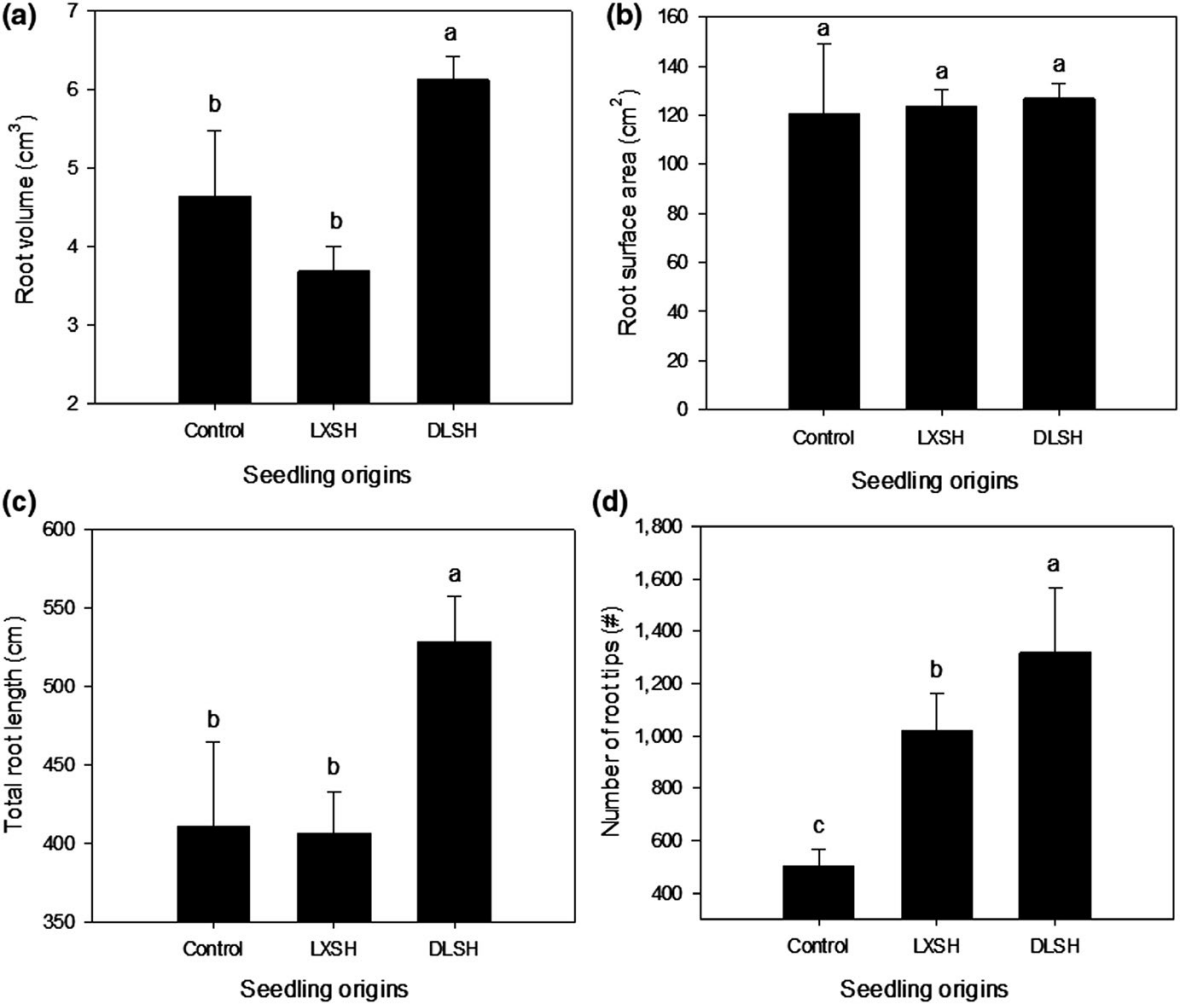
Root volume (a), root surface area (b), total root length (c), and number of root tips of F1 progeny from the DMS population hybridized with pollen collected from the DLS population (DLSH) and LXS population (LXSH). Naturally open‐pollinated DMS plants were selected as the control. The data are presented as mean ± *SD* values. Different lowercase letters indicate significant differences between different pollen sources at a *p* < .05 level

The root volume and total root length of seedlings of DLSH were significantly higher than those of the LXSH and control seedlings (Figure 5a,c). The DLSH seedlings had significantly more root tips than the LXSH seedlings, which had significantly more than the control seedlings (Figure 5d).

Principal component analysis revealed that the morphological performance of DLSH seedlings and LXSH seedlings were similar, and both differed from that of the control seedlings (Figure 3b). Basal diameter and root volume made significant positive and negative contributions to PC1, respectively (Figure 3b).

### 3.3 Photosynthetic physiological performance of seedlings

**Figure 6:**
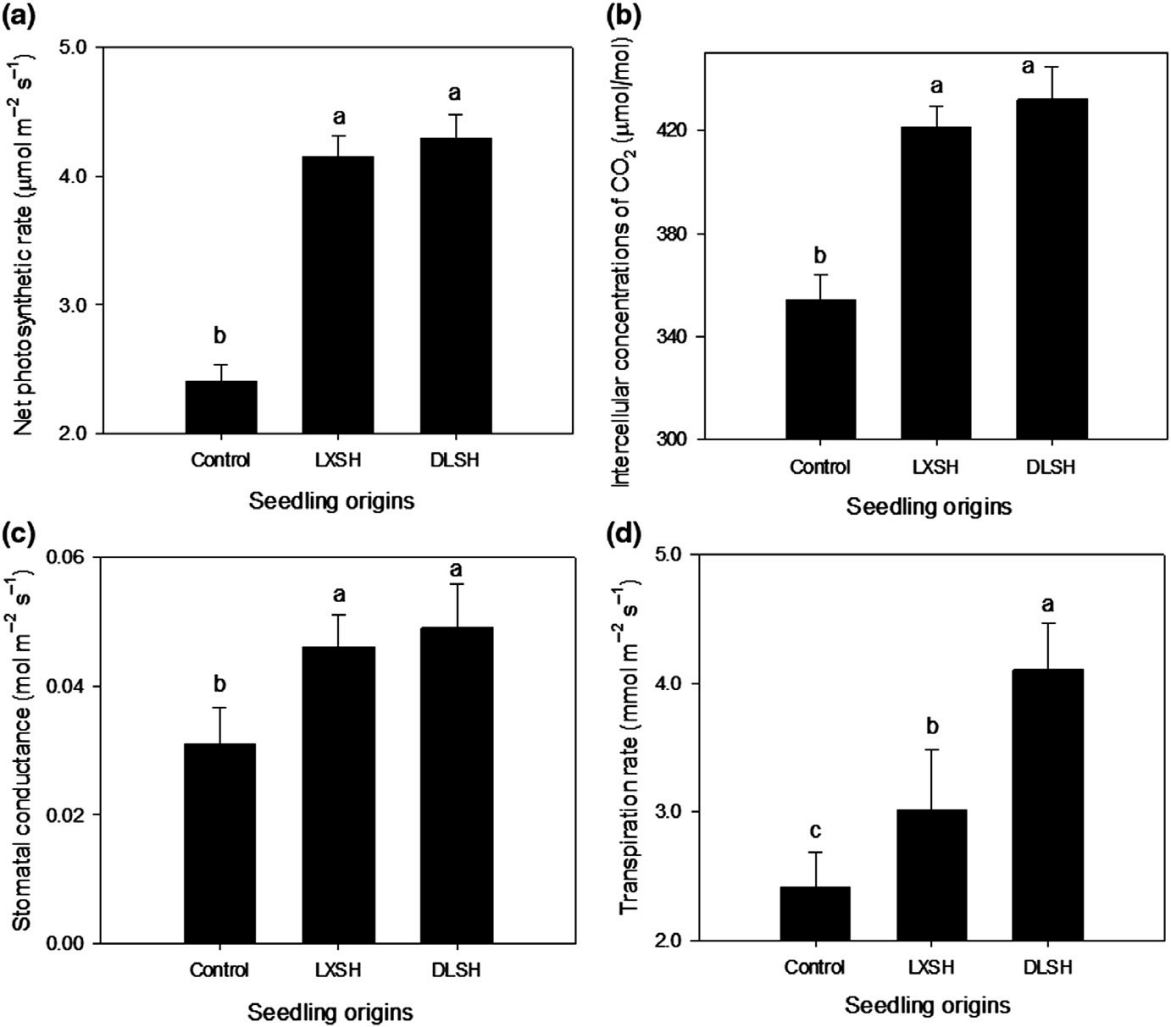
Net photosynthetic rate (a), intercellular concentration of CO_2_ (b), stomatal conductance (c), and transpiration rate of F1 progeny from the DMS population hybridized with pollen collected from the DLS population (DLSH) and LXS population (LXSH). Naturally open‐pollinated DMS plants were selected as the control. The data are presented as mean ± *SD* values. Different lowercase letters indicate significant differences between different pollen sources at a *p* < .05 level

The DLSH and LXSH seedlings had similar *P*_n_, *C*_i_, and *G*_s_ values, which were significantly higher than those of the control seedlings (Figure 6a–c). *T*_r_ values of the DLSH seedlings were significantly higher than those of in the LXSH seedlings, which was significantly higher than those of the control (Figure 6d).

**Figure 7:**
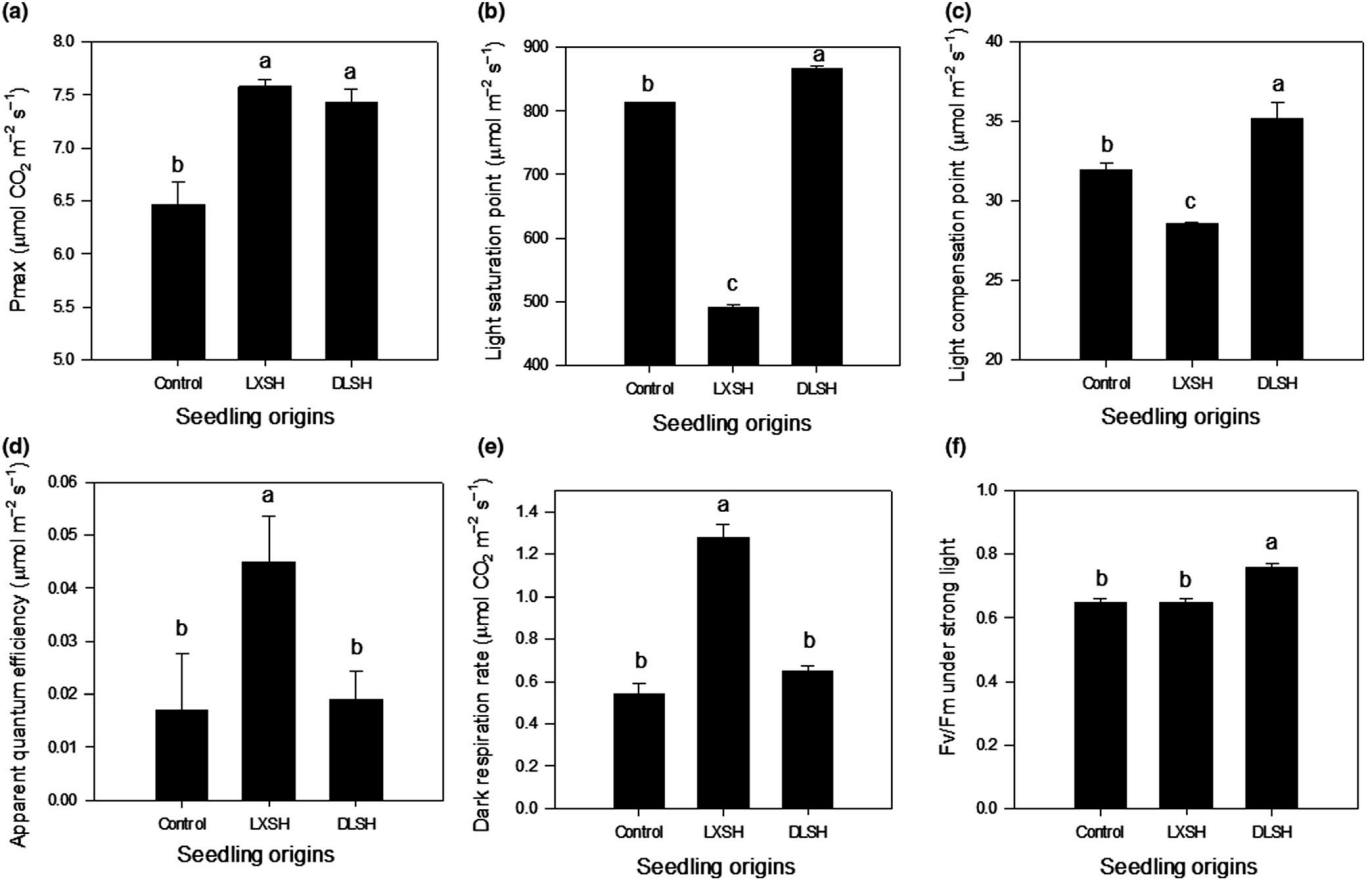
*P*max (a), light saturation point (b), light compensation point (c), apparent quantum efficacy (d), dark transpiration rate (e), and *F*v/*F*m under strong light (f) for F1 progeny from the DMS population hybridized with pollen collected from the DLS population (DLSH) and LXS population (LXSH). Naturally open‐pollinated DMS plants were selected as the control. The data are presented as mean ± *SD* values. Different lowercase letters indicate significant differences between different pollen sources at a *p* < .05 level

The light response curve of control, DLSH, and LXSH seedlings are shown in Figure S1. The DLSH and LXSH seedlings had similar *P*_max_ values, which were significantly higher than that in control seedlings (Figure 7a). LSP and LCP values in the DLSH seedlings were significantly higher than those of control seedlings, which were significantly higher than those of LXSH seedlings (Figure 7b,c). The LXSH and control seedlings had similar *AQY* and *R*_d_ values, which were significantly lower than those of DLSH seedlings (Figure 7d,e).

Under strong light (1,200 μmol photons m^−2^ s^−1^), DLSH seedlings exhibited a *F*_v_/*F*_m_ value of 0.75, while the other two seedlings exhibited a *F*_v_/*F*_m_ value of 0.65 (Figure 7f), indicating the efficiency of DLSH seedlings under strong light.

Principal component analysis showed that the photosynthetic physiological performance varied among DLSH, LXSH, and control seedlings (Figure 3c). LSP, LCP, and *F*_v_/*F*_m_ made significant positive contributions to PC1 (Figure 3c).

### 3.4 Genetic diversity of F1 progeny

**Figure 8:**
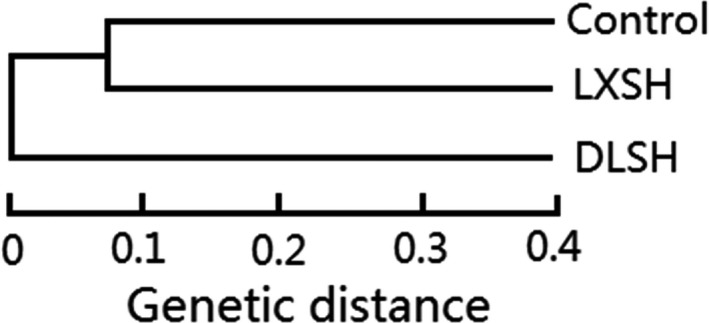
Dendrogram of different F1 progeny produced using the UPGMA cluster method based on SRAP data*.* Control, seedlings from naturally occurring seeds; LXSH, DMS × LXS seeds; DLSH, DMS × DLS seeds. Genetic distance indicates Nei's genetic distance (Nei, 1972) calculated using POPGENE version 1.31 software

Eighty‐eight combinations of SRAP primers were used for PCR amplification, and finally, eight combinations of SRAP primers (me1–em4, me1–em5, me2–em8, me2–em9, me3–em4, me3–em8, me4–em8, me8–em11) that produced clear and reproducible DNA fragments were selected and used for genotyping. A total of 50 dominant loci were amplified with an average of 6.25 loci per primer. Among these, 39 loci (*p* = 78%) were polymorphic. Table 3 summarizes the *h* and *I* values, with averages of 0.2734 and 0.4085, respectively. The genetic diversity of F1 progenies, as estimated by *h* and *I*, placed DLSH as the most diverse, followed by LXSH and, lastly, the control progeny (Table 3). The genetic relationships based on Nei's genetic distance are presented in Figure 8. Control and LXSH progeny were the most genetically similar and clustered into a group first, with DLSH clustering outside of these two sister groups.

**Table 3:** Genetic diversity of F1 progenies of *Sinocalycanthus chinensis* DMS population hybridized with different pollen sources

Hybrids	*N*	*L*	*P* (%)	*I*	*h*
Control	30	16	32.00	0.1544	0.1004
LXSH	30	14	28.00	0.1578	0.1062
DLSH	30	20	40.00	0.2302	0.1569
Total	90	50	78.00	0.4085	0.2743

Note

Control, natural F1 progeny of the DMS population; LXS, F1 progeny of the DMS *S. chinensis* population hybridized with pollen collected from the LXS population; DLSH, F1 progeny of the DMS *S. chinensis* population hybridized with pollen collected from the DLS population; *N*, number of samples; L, number of polymorphic loci; *P*, percentage of polymorphic loci; *I*, Shannon diversity index; *h*, Nei's diversity index.

## 4 DISCUSSION

In this study, outcrossed F1 progeny sired with pollen from two other populations had higher genetic diversity, performance, and fitness than the naturally occurring control progeny, indicating that genetic rescue by introducing unrelated genetic materials can mitigate these effects, thus enhancing the viability of endangered *S. chinensis* populations. As suggested by Li and Jin (2006), the transplantation of individuals from different populations can increase gene flow and the consequent fitness of otherwise inbred offspring; similarly, Li, Jin, and Tan (2012) suggested that transplantation and hand pollination should be used to reduce inbreeding depression. For *S. chinensis*, a species with high genetic differentiation among populations, genetic rescue by interpopulation crosses should be recommended as a useful conservation strategy.

Genetic rescue is effective for endangered plant species with limited gene flow and high genetic differentiation among populations. Finger et al. (2011) found that genetic rescue is effective in *Medusagyne oppositifolia*, a threatened tree species with limited gene flow. Ge et al. (1998) developed a conservation strategy for genetic rescue by conducting crosses between populations with high genetic differentiation in *Cathaya argyrophylla*. *Sinocalycanthus chinensis* is an endangered plant characterized by high genetic differentiation among populations with limited gene flow based on random amplified polymorphism DNA (RAPD, *G*_ST_ = 0.7613, *N*_m_ = 0.1568; Li & Jin, 2006), intersimple sequence repeat (ISSR, *G*_ST_ = 0.5779, *N*_m_ = 0.3651; Li, Jin, & Gu, 2012) and chloroplast microsatellite (cpSSR, *G*_ST_ = 0.7547, *N*_m_ = 0.1616; Li, Jin, & Tan, 2012) markers. *Sinocalycanthus chinensis* is entomophilous and pollinated by small insects that are flightless or have limited flight, which mainly visit flowers of the same individual plant (Li & Jin, 2006). The seeds of *S. chinensis* are surrounded by pericarp, and, when ripe, the seeds are dispersed only by gravity; thus, the dispersion of offspring is quite limited (Li & Jin, 2006). In addition, *S. chinensis* is self‐compatible, which also consequently limits gene flow among populations (Zhang & Jin, 2008; Zhao et al., 2011). All the above factors contribute to the maintenance of high population differentiation, thereby resulting in the efficacy of genetic rescue in this species.

Pollen sources can influence the offspring fitness and the consequent population viability (Aguilar, Ashworth, Galetto, & Aizen, 2006). González‐Varo et al. (2010) evaluated the genetic diversity, mating patterns, and progeny performances in fragmented populations of the Mediterranean shrub *Myrtus communis*, finding that it was possible to enhance the fitness and genetic diversity of progeny by promoting outcrossed mating among fragmented populations of the self‐compatible species. Similar to *M. communis*, *S. chinensis* is an insect‐pollinated shrub with populations occurring across an extremely fragmented landscape. In this study, the genetic diversity (Table 3), fitness, and performance (Figure 2) of DLSH and LXSH progeny were generally higher than those of control progeny, revealing the efficacy of genetic rescue. These results also determined that DLS would be the optimal pollen parent population. DLSH F1 progeny had the highest fitness, the strongest morphological and physiological performance, and the highest genetic diversity.

In most species with multi‐seeded fruits, seeds apparently compete for limited maternal resources, resulting in a trade‐off between seed size and seed number (Baker et al., 1994; Lyons & Antonovics, 1991). In this study, crosses of DMS with pollen from DLS populations produced the most seeds per fruit and the highest thousand‐seed weight, indicating that pollen sources play an important role in the reproductive success of *S. chinensis* (Baker et al., 1994). Larger seeds often tend to germinate earlier and more reliably, and seedlings from large seeds are more likely to survive (Stanton, 1985). The DLSH seeds germinated later but had higher germination rates and seedlings emergence rates than those of LXSH and control seeds, indicating genetic rescue can enhance progeny fitness by introducing novel genetic material. In the PCA, the contributions of total germination rate, seedlings emergence rate, and germination days to PC1 were associated with the differences in fitness of DLSH and LXSH progeny from that of the control progeny (Figure 3a), indicating the outcrossed F1 progeny had higher fitness, that is, they germinated quicker and more often.

In natural populations, plant growth and other morphological traits are positively correlated with components of fitness (Molina‐Montenegro et al., 2013). Root morphological traits, such as total root length and volume, indicate the distribution of roots in the soil profile and amount of water absorbed (Ludlow & Muchow, 1990). A larger root system (with greater lateral branching and rooting depth) has been associated with increased transpiration, shoot biomass production, and harvest index values through the ability of plants to use more soil moisture (Vadez, Kholova, Medina, Kakkera, & Anderberg, 2014). Although few studies have tested the correlation between root morphological traits and fitness as a consequence of the obvious difficulties presented by root phenotyping (Sofi, Djanaguiraman, Siddique, & Prasad, 2018), associations of fitness with root length and volume have exhibited a range of conclusions (for positive associations, see Bishopp & Lynch, 2015; Sofi et al., 2018; for negative associations or no association, see Vadez et al., 2014; Purushothaman, Krishnamurthy, Upadhyaya, Vadez, & Varshney, 2017). In this study, we found that the DLSH seedlings had the strongest growth performance, including plant height, basal diameter, root volume, total root length, and number of root tips, and consequently had the highest fitness, indicating a positive association. In the PCA, there were positive and negative contributions of basal diameter and root volume to PC1, respectively, corresponding to the morphological traits of DLSH and LXSH progeny differing from those of the control progeny (Figure 3b) and indicating the outcrossed F1 progeny invested more resources in aboveground growth rather than belowground growth.

Moreover, we have also found that DLSH seedlings had the highest photosynthetic indices, including *P*_n_, *C*_i_, *G*_s_, *T*_r_, *P*_max_, LSP, and LCP, and consequently had the highest fitness. A higher photosynthesis rate leads to increased resource uptake, which can be allocated to different plant functions, thus improving growth, fecundity, and survival (Arntz, DeLucia, & Jordan, 2000; Molina‐Montenegro et al., 2013). Molina‐Montenegro et al. (2013) suggest that the maximum quantum yield of PSII (i.e., *F*_v_/*F*_m_) seems to be a good predictor of plant fitness because even minor changes in plant physiological status can have direct and significant consequences on plant reproduction. The value of *F*_v_/*F*_m_ is almost constant for different plant species measured under nonstressful conditions (0.8 < *F*_v_/*F*_m_ < 0.86) (Björkman & Demmig, 1987). Chai et al. (2012) found that *F*v/*F*m was an indicator of plant’ fitness under exposure to high‐intensity light. *F*_v_/*F*_m_ declined dramatically under high‐intensity light (Nakamura, Hidema, Sakamoto, Ishida, & Izumi, 2018). In this study, we found that DLSH seedlings exhibited a *F*_v_/*F*_m_ value of 0.75 under strong light, while DLSH and control seedlings had only a value of 0.65, indicating a wide adaptability of DLSH seedlings across a range of variation in environmental factors. In the PCA, the positive contributions of LSP, LCP, and *F*_v_/*F*_m_ to PC1 were associated with the photosynthetic characteristics of DLSH progeny differing from those of control and LXSH progeny (Figure 3c), indicating the F1 progeny of DLSH shared the high‐intensity light adaptive ability of the control, which plays an important role in progeny fitness.

In this study, we found that DLS pollen donors were superior to LXS pollen donors, which might be affected by the genetic and environmental effects of the pollen sources populations. The genetic pool of pollen parents affects the genetic diversity of progeny (Zhu & Li, 2010). In this study, pollen from the DLS population, which has relatively higher genetic distance from the DMS population (Nei's genetic distance = 0.2034, Gu, 2010) produced F1 progeny with relatively higher genetic diversity (*I* = 0.2302; *h* = 0.1569), while LXS pollen, typified by relatively lower genetic distance from the DMS population (Nei's genetic distance = 0.1027, Gu, 2010), produced F1 progeny with relatively lower genetic diversity (*I* = 0.1578; *h* = 0.1062). Zhu and Li (2010) analyzed the genetic diversity of *Liriodendron chinense* progeny produced from crosses with different pollen sources and found that progeny had higher genetic diversity than progeny produced by selfing and observed a positive relationship between genetic diversity and the genetic distance between parents. Although studies assessing progeny performance are scarce, the genetic pool of parents can affect the performance of progeny (Pickup et al., 2013; Yates et al., 2007).

Geographic distance has very little power to predict hybrid progeny fitness for any of the fitness components or across generations (Pickup et al., 2013). In our study, pollen from the DLS population, which is geographically distant from the DMS population (209.128 km), produced F1 progeny with relatively higher genetic diversity, relatively higher performance, and relatively higher fitness, while pollen from the LXS population, which is geographically close to the DMS population (27.345 km), produced F1 progeny with relatively lower genetic diversity, relatively lower performance, and relatively lower fitness, indicating that geographical distance might be associated with the genetic distance separating populations and, in turn, the fitness of resulting progeny. Although there were significant genetic isolation and a significantly positive relationship between geographic distance and genetic distance among populations of *S. chinensis* (Li, Jin, & Gu, 2012; Li, Jin, & Tan, 2012), further studies are needed to verify the possible association.

The genetic diversity, morphological and photosynthetic physiological traits, and fitness of F1 progeny suggest that genetic rescue with pollen from the DLS population would be effective in the conservation of endangered *S. chinensis*, which exhibits high genetic differentiation among populations and limited gene flow among populations. Although it is well known that genetic rescue by transfer of genetic materials from different populations would benefit the species, it may be dependent on the reproductive traits and genetic variation in the species. Based on the results in this study, we suggest that genetic rescue by transplanting seedlings from different populations or artificial hand pollination with pollen from different population would be an effective method for reducing inbreeding depression and obtaining vigorous offspring with high genetic diversity and fitness in endangered *S. chinensis*. Although the results indicate that geographical and genetic distances between seed parents played a key role in the genetic rescue of *S. chinensis*, we could not rigorously test the correlations of genetic diversity with fitness, photosynthetic performance, and morphological performance, as there were only two populations used in the experiment as pollen parents. Further studies should use more populations to infer this relationship and similar species in order to provide broader reference data for genetic rescue.

## CONFLICT OF INTEREST

The authors declare no conflict of interest.

## AUTHOR CONTRIBUTIONS

**Junmin Li:** Conceptualization (supporting); funding acquisition (supporting); methodology (supporting); project administration (supporting); resources (supporting); supervision (supporting); validation (equal), visualization (equal), writing – original draft (lead), writing – review and editing (lead). **Caihong Qi:** Data curation (equal), formal analysis (equal), investigation (equal), methodology (equal), writing – review and editing (supporting). **Jingjing Gu:** Data curation (equal), formal analysis (equal), investigation (equal), methodology (equal), writing – review and editing (supporting). **Zexin Jin:** Conceptualization (lead), funding acquisition (lead), project administration (lead), resources (lead), supervision (lead), validation (equal), visualization (equal), writing – review and editing (supporting).

## Supporting information

Figure S1

Figure S1_caption

## Data Availability

The data that support the findings of this study have been deposited in Dryad with https://doi.org/10.5061/dryad.stqjq2c0h.
