# Supplementary figures and images for: Effect of sire population on the genetic diversity and fitness of F1 progeny in the endangered Chinese endemic Sinocalycanthus chinensis
Source: Ecol Evol. 2020 Apr 3;10(9):4091–103. doi: 10.1002/ece3.6179 (PMC7244809; doi:10.1002/ece3.6179)

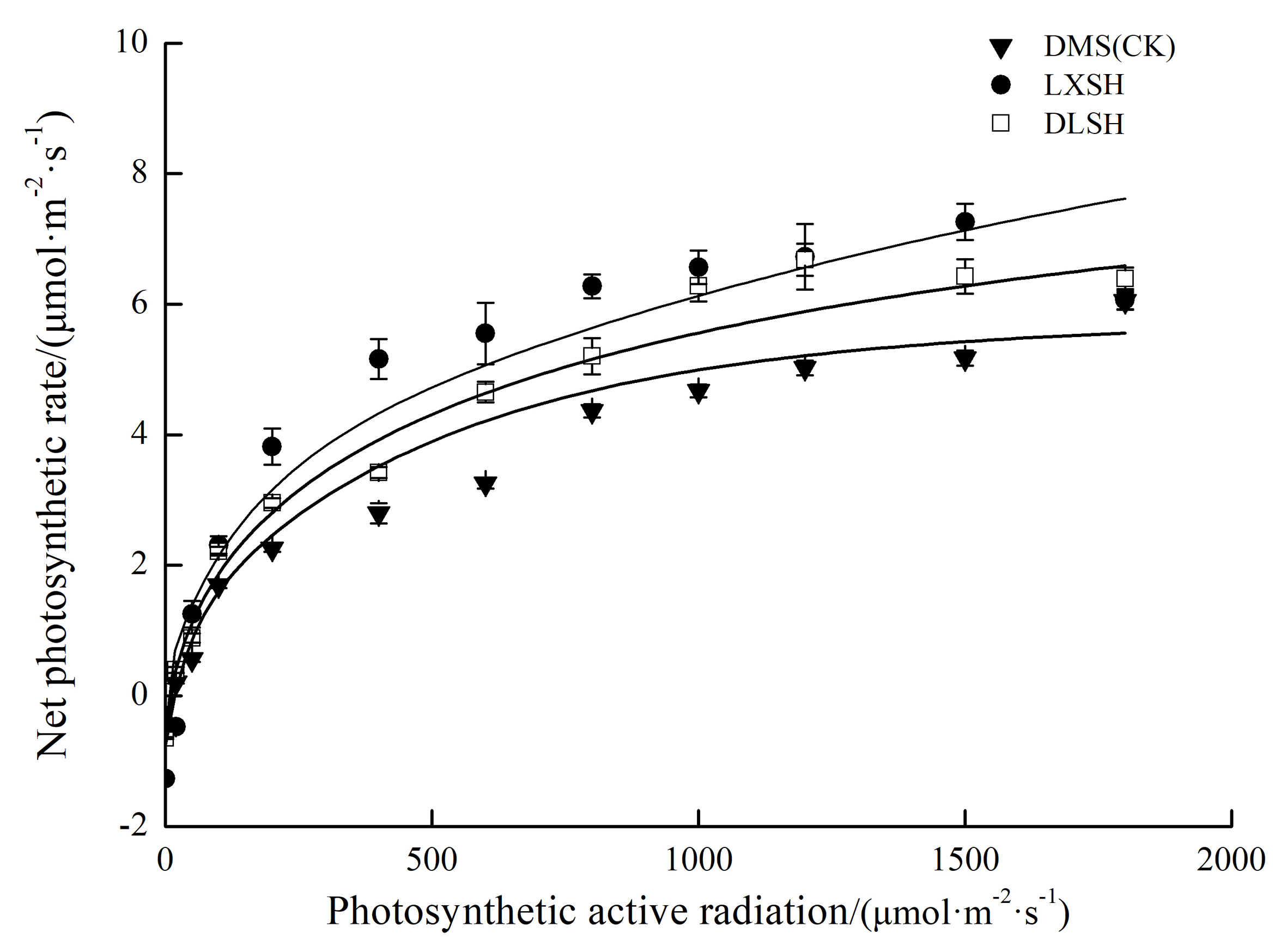

Supplement: Supplementary file 1 — Figure S1 [file ECE3-10-4091-s001.tif]
