# Supplementary material for: Effect of sire population on the genetic diversity and fitness of F1 progeny in the endangered Chinese endemic Sinocalycanthus chinensis
Source: Ecol Evol. 2020 Apr 3;10(9):4091–103. doi: 10.1002/ece3.6179 (PMC7244809; doi:10.1002/ece3.6179)
Supplement: Supplementary file 2 — Figure S1_caption [file ECE3-10-4091-s002.docx]

**Supporting materials**

Fig. S1 The light response curves of F1 progeny from the DMS population hybridized with pollen collected from the DLS population (DLSH) and LXS population (LXSH). Naturally open-pollinated DMS plants were selected as the control (CK). The data are presented as mean ± standard deviation values.
